# Brain structural differences in children with fetal alcohol spectrum disorder and its subtypes

**DOI:** 10.3389/fnins.2023.1152038

**Published:** 2023-08-09

**Authors:** Theresah Boateng, Kathryn Beauchamp, Faerl Torres, Chaselyn D. Ruffaner-Hanson, John F. L. Pinner, Kishore Vakamudi, Cassandra Cerros, Dina E. Hill, Julia M. Stephen

**Affiliations:** ^1^Department of Special Education, The University of New Mexico, Albuquerque, NM, United States; ^2^College of Pharmacy, University of New Mexico Health Sciences Center, Albuquerque, NM, United States; ^3^The Mind Research Network, Division of the Lovelace Biomedical Research Institute, Albuquerque, NM, United States; ^4^Department of Neurosciences, University of New Mexico Health Sciences Center, Albuquerque, NM, United States; ^5^Department of Pediatrics, University of New Mexico Health Sciences Center, Albuquerque, NM, United States; ^6^Department of Psychiatry and Behavioral Sciences, University of New Mexico Health Sciences Center, Albuquerque, NM, United States

**Keywords:** brain volume, fetal alcohol spectrum disorders, magnetic resonance imaging, prenatal alcohol exposure, children

## Abstract

**Introduction:**

The teratogenic effects of prenatal alcohol exposure (PAE) have been examined in animal models and humans. The current study extends the prior literature by quantifying differences in brain structure for individuals with a fetal alcohol spectrum disorder (FASD) compared to typically developing controls, as well as examining FASD subtypes. We hypothesized the FASD group would reveal smaller brain volume, reduced cortical thickness, and reduced surface area compared to controls, with the partial fetal alcohol syndrome (pFAS)/fetal alcohol syndrome (FAS) subtypes showing the largest effects and the PAE/alcohol-related neurodevelopmental disorder (ARND) subtype revealing intermediate effects.

**Methods:**

The sample consisted of 123 children and adolescents recruited from a single site including children with a diagnosis of FASD/PAE (26 males, 29 females) and controls (34 males, 34 females). Structural T1-weighted MRI scans were obtained on a 3T Trio TIM scanner and FreeSurfer v7.2 was used to quantify brain volume, cortical thickness, and surface area. Analyses examined effects by subgroup: pFAS/FAS (*N* = 32, M_age_ = 10.7 years, SE_age_ = 0.79), PAE/ARND (*N* = 23, M_age_ = 10.8, SE_age_ = 0.94), and controls (*N* = 68, M_age_ = 11.1, SE_age_ = 0.54).

**Results:**

Total brain volume in children with an FASD was smaller relative to controls, but subtype analysis revealed only the pFAS/FAS group differed significantly from controls. Regional analyses similarly revealed reduced brain volume in frontal and temporal regions for children with pFAS/FAS, yet children diagnosed with PAE/ARND generally had similar volumes as controls. Notable differences to this pattern occurred in the cerebellum, caudate, and pallidum where children with pFAS/FAS and PAE/ARND revealed lower volume relative to controls. In the subset of participants who had neuropsychological testing, correlations between volume and IQ scores were observed. Goodness-of-Fit analysis by age revealed differences in developmental patterns (linear vs. quadratic) between groups in some cases.

**Discussion:**

This study confirmed prior results indicating decreased brain volume in children with an FASD and extended the results by demonstrating differential effects by structure for FASD subtypes. It provides further evidence for a complex role of PAE in structural brain development that is likely related to the cognitive and behavioral effects experienced by children with an FASD.

## 1. Introduction

Fetal alcohol spectrum disorders (FASDs) constitute a range of conditions from mild to severe and can include deficits in cognition and behavior as well as neurological and physical abnormalities as a result of prenatal alcohol exposure (PAE). Fetal alcohol syndrome (FAS), partial fetal alcohol syndrome (pFAS), and alcohol-related neurodevelopmental disorder (ARND) constitute the component subtypes of FASD (Hoyme et al., 2016). Physical effects, cognitive deficits, and behavioral challenges, and concomitant social and adaptive functioning difficulties have been documented as sequelae of PAE (Franklin et al., 2008; Mattson et al., 2013; Williams and Smith, 2015). A significant percentage of children with an FASD, especially those with an ARND subtype, are not diagnosed, under-diagnosed, or misdiagnosed due to the focus on craniofacial anomalies, confusion with diagnostic terminologies, and attribution of symptoms to other disorders, such as attention deficit hyperactivity disorder (ADHD) (Aase, 1994; Astley, 2010; Chasnoff et al., 2015; Hoyme et al., 2016). The Center for Disease Control and Prevention (CDC) has estimated the prevalence of FASD in the United States to be in the range of 1–5 per 100 school children (Sharma, 2001; May et al., 2009, 2014, 2018), which indicates a need for further understanding of the effects of PAE on brain development.

Magnetic resonance imaging (MRI) studies of individuals with PAE have identified structural changes in brain size and shape (including microcephaly), gray and white matter volumes, cortical thickness (CT), and surface area (SA) in children, adolescents, and young adults (Sowell et al., 2002b; Riley et al., 2004; Lebel et al., 2011; Zhou et al., 2011; Roussotte et al., 2012; Yang et al., 2012a). While the severity of abnormalities is positively correlated with higher doses of PAE (Sowell et al., 2008; Zhou et al., 2011; Hoyme et al., 2016; Marshall et al., 2022), some studies found teratogenic effects due to PAE even in cases of minimal alcohol consumption in humans (Joseph et al., 2014; Williams and Smith, 2015; Marshall et al., 2022).

The corpus callosum, cerebellum, and subcortical regions, including the hippocampus and basal ganglia, have consistently emerged as significantly affected due to PAE in earlier autopsy and imaging studies (Jones et al., 1973; Riley et al., 2004). For example, Jacobson et al. (2017) reported significantly smaller corpus callosum in infants subsequently diagnosed with FAS (*n* = 43) relative to controls. Previous studies revealed total brain volume reductions in gray and white matter in individuals with PAE relative to controls (Archibald et al., 2001; Inkelis et al., 2020), with reports of total brain volume decreases in the pFAS/FAS groups (Archibald et al., 2001; Treit et al., 2016). In addition, Sowell et al. (2002b) showed regional increases in gray matter (GM) volumes in FASD participants. Similarly, Treit et al. (2014) reported less cortical thinning with age in children with FASD. In contrast, reports from Zhou et al. (2018) revealed volume reductions in all brain regions among the PAE group, where total GM in PAE showed a negative correlation with age. These inconsistent reports of the teratogenic effects of PAE on brain volume may be due to variability in dosage, duration, and timing of alcohol exposure, genetics, maternal nutrition, and maternal and fetal metabolism (Petrelli et al., 2018).

Regional volume analyses indicated that inferior parietal/perisylvian regions and the anterior and orbital frontal cortices were significantly reduced in individuals with PAE relative to the control groups (Archibald et al., 2001; Sowell et al., 2002a). However, parietal lobes showed greater reductions than the frontal and temporal lobes in human studies (Archibald et al., 2001; Sowell et al., 2002a; Fernández-Jaén et al., 2011).

While brain volume encompasses both SA and CT, these measures may differ independently, providing additional insight into the underlying mechanisms related to PAE-induced brain damage. PAE-related SA decreases were reported (Migliorini et al., 2015), even within ARND sub-types. However, right temporal lobe SA only approached significance in the ARND groups in Rajaprakash et al. (2014), and the SA analysis performed by Migliorini et al. (2015) was limited to the anterior cingulate cortex. Thus, there is limited knowledge regarding PAE effects on SA throughout the brain.

There are reports of decreased CT among individuals with PAE compared to controls in children (Robertson et al., 2016), children and adolescents (Zhou et al., 2018), and adolescents and adults (Zhou et al., 2011), including reduced CT in the PAE group relative to controls in bilateral regions of frontal, temporal, parietal, and occipital cortices; there was no evidence of increased CT in PAE. In contrast, other studies reported increased CT among the PAE groups relative to controls in children (Treit et al., 2014), children and adolescents (Fernández-Jaén et al., 2011), and in children, adolescents, and young adults (Sowell et al., 2008). Some of these differences may be related to differences in age across these studies.

The most prominent reductions in brain morphology were evident in pFAS/FAS groups (i.e., individuals with dysmorphic physical features) relative to the PAE/ARND and control groups (Roussotte et al., 2012; Yang et al., 2012b). Individuals diagnosed with FAS had the most severe agenesis and general malformations in the brain (Sowell et al., 2002a; Riley et al., 2004; Lebel et al., 2011; Yang et al., 2012a). However, few studies have directly compared FASD subtypes with respect to volume, CT, and SA across age categories (infants, children, adolescents, young adults, and adults) or sex, although, to the best of our knowledge, all human studies examining brain structure in FASD included male and female participants.

Animal models have shown similar results (O’Leary-Moore et al., 2011; Coleman et al., 2012; Leigland et al., 2013; Zhang et al., 2019) providing additional evidence of the robustness and generalizability of PAE effects on brain structure. These similarities include overall decreases in brain volume (Coleman et al., 2012; Leigland et al., 2013) and dose effects with greater structural abnormalities associated with higher doses of alcohol (Leigland et al., 2013; Zhang et al., 2019). Similar to human studies, there are some conflicting reports of the effects of PAE on brain volume. Coleman et al. (2012) reported decreased brain volume in mice with PAE, while Zhang et al. (2019) revealed no effect of ethanol exposure on whole brain volume in mice. Regional analysis largely focused on frontal and hippocampal regions, with only recent explorations of the functional alterations in parietal lobe (O’Leary-Moore et al., 2011; Leigland et al., 2013; Wang and Kroenke, 2015). PAE-related SA decreases (Leigland et al., 2013) and CT decreases (O’Leary-Moore et al., 2011; Coleman et al., 2012; Leigland et al., 2013; Wang and Kroenke, 2015) were reported in mice. Animal models provide important information regarding metrics that are difficult to control in human studies including exposure amounts to prenatal alcohol. However, large differences in overall brain structure, alcohol metabolism rates and developmental trajectories lead to some challenges in direct translation between animal and human studies.

The purpose of the current study was to examine structural brain differences in children with an FASD; specifically, to investigate effects in FASD subtypes versus control children in a relatively large sample collected at a single site. Structural MRIs (sMRIs) were collected in conjunction with our ongoing MEG studies for source analysis (Stephen et al., 2012; Coffman et al., 2013). We hypothesized that children with an FASD would show smaller brain volume, reduced CT, and reduced SA compared to controls with regionally specific deficits and differential effects in those with and without dysmorphic features due to PAE resulting in the smallest volumes in the pFAS/FAS subtype, mid-level values in the PAE/ARND subtype, and the largest volume measurements in controls. The examination of structural brain metrics in this cohort allowed us to examine if brain structure differs between FASD subtypes potentially indicating differences between those with and without dysmorphic features.

## 2. Materials and methods

### 2.1. Participants

**FIGURE 1:**
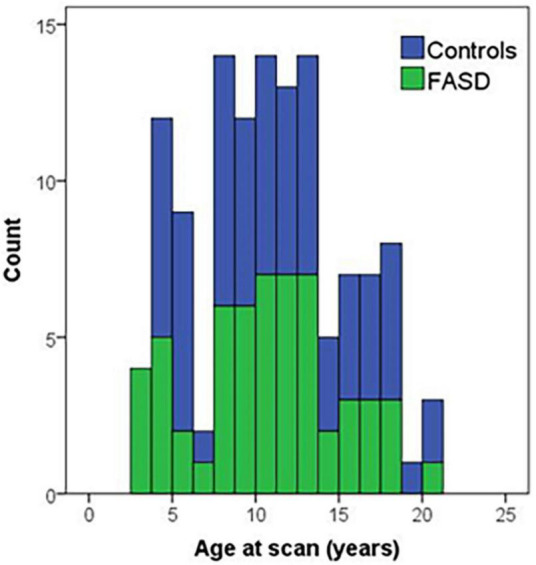
Histogram of age of participants at the time of the MRI scan by group. Controls are in blue and FASD is shown in green.

**FIGURE 2:**
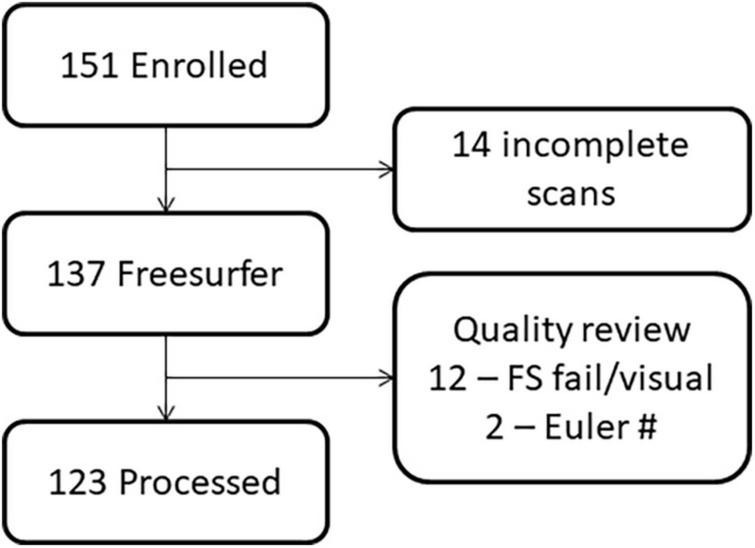
Flowchart demonstrating the loss of participants due to challenges in imaging young children versus data quality.

The study procedures and human subjects’ protocol were approved by the University of New Mexico Health Sciences Center Human Research Review Committee (UNMHSC HRRC). All parents were consented and children ≥7 years of age were assented prior to study participation. Participants were recruited under a single Internal Review Board protocol designed to understand the effects of PAE on brain development. Children from different age ranges (3–7, 8–12, and 12–21 years) were recruited across a 10-year period, representing different cohorts and different cognitive assessments appropriate for age. Sex was self-reported by the parent/participant. Data were collected between 2010 and 2019. Of 151 individuals (total number of children with FASD and neurotypical controls) initially enrolled in the MRI portion of the study, we successfully collected a T1 MRI sequence from 137 children. Of these, 123 sMRIs were of sufficient quality (after excluding *N* = 14 due to subject motion or other artifact) to undergo auto-analysis processing through the FreeSurfer pipeline to allow for volume quantification (see Figures 1, 2).

Children with an FASD and PAE were recruited from the FASD clinic located within the Center for Development and Disability, a University Center for Excellence in Developmental Disabilities Education, Research, and Service housed within the Pediatrics Department of the University of New Mexico Health Sciences Center. Diagnosis on the FASD continuum was based on consensus from an interdisciplinary assessment team comprised of a clinical psychologist, neuropsychologist, and pediatrician using the Institute of Medicine guidelines and the revised guidelines (Stratton et al., 1996; Hoyme et al., 2016). Children with a differential diagnosis due to a genetic disorder or exposure to other teratogens were excluded. Maternal alcohol consumption was confirmed through either maternal interview, eyewitness reports of maternal drinking during pregnancy, or legal records documenting alcohol consumption during pregnancy (e.g., birth records or recorded incident of driving while intoxicated). Information on maternal alcohol consumption during pregnancy was collected as part of the FASD clinical assessment; however, specific estimates of quantity of alcohol consumption during pregnancy were generally not available. PAE was defined to include documentation of ≥6 drinks per week for ≥2 weeks during pregnancy, ≥3 drinks per occasion on ≥2 occasions during pregnancy, intoxication or history of treatment of an alcohol-related condition, and an assessment using screening tools. Children diagnosed with FAS met the following criteria: at least two of three characteristic facial anomalies, growth restriction and/or deficient brain growth, and cognitive and/or behavioral impairments. Children diagnosed with pFAS with known PAE met the following criteria: at least two of three characteristic facial anomalies and cognitive and/or behavioral functioning impairments. Children diagnosed with pFAS without confirmed PAE met the following criteria: at least two of three characteristic facial anomalies, deficient brain growth, and cognitive and/or behavioral functioning impairments. Total number of participants with FAS (*N* = 25) and pFAS (*N* = 7) was 32. Lastly, children diagnosed with ARND (*N* = 15) met the following criteria: age 3 years or older, confirmation of PAE, and cognitive and/or behavioral functioning impairments. The PAE participants (*N* = 8) were recruited from the FASD clinic and met criteria for PAE without meeting all criteria demonstrating cognitive/behavioral effects. The control participants (*N* = 68) were recruited from the community on the basis of reported absence of PAE and other prenatal exposures including tobacco and illicit substances (including cannabis) based on parental interview, no known neurodevelopmental disorder, and, if tested, IQ scores > 70.

### 2.2. Neuropsychological/cognitive assessment

Depending on the study cohort, participants were provided neuropsychological testing using the Wechsler Abbreviated Scale of Intelligence second edition (WASI-II Full Scale) to assess IQ scores. The WASI-II was not collected for some children between 3 and 11 years of age (some data were collected as a part of pilot data collection that did not include neuropsychological testing), such that the number of participants with reported IQ scores was 86 (40 FASD and 46 controls). Similarly, a subset of children (*N* = 71; 25 FASD and 46 controls) was assessed using the Grooved Pegboard (GPB) test to measure fine motor function. Because the data were collected in age- and sex-matched cohorts the cognitive testing was applied equally across groups.

### 2.3. Structural MRI (sMRI) data acquisition

This analysis included participants who were recruited into three different studies across a 10-year period who underwent a sagittal T1-weighted anatomical MRI scan in a Siemens TIM Trio 3 Tesla MRI system with a five-echo 3D MPRAGE sequence [TR/TE/TI - 2530/1.64, 3.5, 5.36, 7.22, 9.08/1200 ms, flip angle - 7 deg, field of view (FOV) 256 × 256 mm, 1 mm thick slice, 192 slices, GRAPPA acceleration factor - 2]. Over the 10-year data collection period, a single change in the sMRI imaging protocol occurred with an upgrade from a 12-channel to a 32-channel head coil. Since children were recruited in age-matched cohorts, groups (FASD vs. controls) were well-matched for use of 12- or 32-channel head coils. FreeSurfer version 7.2 (Dale et al., 1999) was used to perform cortical reconstruction and volume segmentation.^1^ The FreeSurfer pipeline included motion correction (Reuter et al., 2010), segmentation, and intensity normalization. All MRIs were visually checked for data quality. For MRIs that showed some motion artifact, cortical parcelation was verified visually following Freesurfer processing. Finally, the Euler number, which is a quantitative approach to evaluate motion (Rosen et al., 2018), was extracted following the Freesurfer processing for each MRI; two participants (1 PAE, 1 FAS) were excluded for being statistical outliers (outside the 3rd quartile ± 1.5 × interquartile range). The statistical analysis focused on total brain volume, CT, SA, region of interest analysis (cerebellum, thalamus, caudate, putamen, pallidum, hippocampus, and amygdala), and lobar analysis (frontal, temporal, parietal, occipital) defined by the Desikan-Killiany FreeSurfer atlas.

### 2.4. Statistical analyses

The final output from the FreeSurfer analysis was transferred to SPSS v.20 for statistical analysis. Given previous research identifying the cerebellum, thalamus, caudate, putamen, pallidum, hippocampus, and amygdala as regions most impacted by FASD, we conducted region of interest (ROI) analyses of these regions specifically. To compare with prior studies, we also examined FreeSurfer generated estimates of frontal, temporal, parietal, and occipital volumes. For all but the corpus callosum and subcortical structures, repeated measures analyses across hemispheres were performed to identify hemispheric differences and interactions between group and hemisphere. We also explored potential differences in CT and SA in the frontal, parietal, temporal, and occipital lobes.

One-way and repeated measures ANOVAs were used to assess two sets of group differences, FASD vs. control, and pFAS/FAS vs. PAE/ARND vs. control, for demographic, behavioral, and sMRI variables (volume, CT, SA). All variables were evaluated for outliers. There were four participants with outlier data representing nine data points identified from the Freesurfer variables (participant 1: left and right parietal SA, right occipital SA, right occipital gray matter (GM) volume; participant 2: left hippocampus, left and right amygdala; and participants 3&4: corpus callosum). Volumes of individual structures were evaluated with and without estimated total intracranial volume (eTIV) as a covariate to determine if the volume was affected similarly relative to the intracranial volume across groups. For regional volume analyses, only analyses with eTIV as a covariate were performed to determine if there were region-specific reductions. One-way ANCOVAs were used for the structures that were not examined by hemisphere (brain segmentation w/out ventricles, eTIV, corpus callosum) including group and sex as between-subjects factors and coil channel count as a covariate of no interest. Repeated measures ANCOVA analyses using the general linear model were used to conduct ROI analyses with hemisphere as a within-subjects factor, group and sex as between-subjects factors, coil channel count as a covariate of no interest, and eTIV as a covariate for volume and SA analyses for all regions. Partial correlations were used to conduct exploratory analyses investigating the association between volume and cognitive measures (IQ and GPB), controlling for age and examining the effects when eTIV was included as an additional covariate. GPB time to completion was used for the partial correlation to match with the un-corrected brain volume data. Only individuals > 9 years of age were included in the GPB correlations due to the change in testing instructions at 9 years of age. No children under 8 years of age received the GPB testing, so elimination of only 8-year-olds resulted in a loss of eight datapoints. For ANOVAs, we controlled for 25 comparisons (accounting for all variables included in Table 1) in the FDR correction and used a *q* = 0.05. For correlations, we only examined correlations with brain volume, limiting the number of comparisons to 13. Corrected *p*-values are reported based on the Benjamini-Hochberg correction (Benjamini and Hochberg, 1995).

**TABLE 1:** Mean (M) values and standard errors (SE) of volume, cortical thickness, and surface area.

	Control		FASD		PAE/ARND	pFAS/FAS
**Volume (mm^3^)**	**M (SE)**	**Age**	**M (SE)**	**Age**	**M (SE)**	**M (SE)**
Total brain vol w/o ventricles	1153384 (15036)	Quad	1036993 (16345)	Quad	1127036 (23822)	960852 (20404)
eTIV	1508244 (21401)	Linear	1370717 (23406)	Linear	1494456 (32963)	1278524 (28233)
Cortical GM	271068 (3514)	Quad	246039 (3844)	Quad	267354 (5380)	230513 (4608)
Corpus callosum vol	3404 (70)	Linear	3013 (75)	Quad	3207 (112)	2860 (97)
Cerebellum cortex vol	54492 (639)	Quad	48071 (699)	Linear	50843 (1019)	45955 (872)
Thalamus vol	7814 (107)	Linear	6946 (117)	Linear	7614 (161)	6444 (138)
Caudate vol	3901 (61)	n/a	3201 (67)	n/a	3550 (95)	2942 (82)
Putamen vol	5229 (81)	Linear	4690 (88)	n/a	5058 (129)	4415 (111)
Pallidum vol	2042 (32)	Linear	1745 (35)	Quad	1921 (50)	1614 (43)
Hippocampus vol	4103 (58)	Linear	3668 (64)	Quad	3924 (94)	3473 (81)
Amygdala vol	1750 (25)	Linear	1583 (27)	Linear	1730 (38)	1471 (33)
Frontal vol	101105 (854)	Quad	97664 (936)	Quad	99033 (1382)	96600 (1330)
Parietal vol	70723 (916)	Linear	70079 (1003)	n/a	73236 (1460)	67230 (1405)
Temporal vol	57820 (535)	Quad	55499 (586)	Quad	56067 (872)	55088 (839)
Occipital vol	25845 (401)	Linear	24651 (444)	n/a	25256 (657)	24133 (624)
**Cortical thickness (CT) (mm)**						
Mean CT	2.673 (0.014)		2.651 (0.016)		2.653 (0.024)	2.650 (0.021)
Frontal CT	2.796 (0.015)		2.790 (0.017)		2.793 (0.026)	2.789 (0.022)
Parietal CT	2.560 (0.014)		2.558 (0.015)		2.568 (0.024)	2.551 (0.020)
Temporal CT	2.983 (0.017)		2.907 (0.018)		2.922 (0.029)	2.900 (0.025)
Occipital CT	2.050 (0.020)		2.024 (0.022)		2.017 (0.034)	2.031 (0.030)
**Surface area (SA) (mm^2^)**						
Total SA	84867 (526)		84676 (576)		85596 (854)	83924 (822)
Frontal SA	30878 (242)		30264 (265)		30381 (394)	30212 (380)
Parietal SA	24148 (209)		24295 (231)		24984 (335)	23682 (318)
Temporal SA	16502 (125)		16293 (137)		16291 (205)	16319 (197)
Occipital SA	11144 (127)		11178 (141)		11266 (210)	11107 (199)

Due to the broad age range of our participants (3–20 years), we conducted a Goodness-of-Fit analysis to determine if age was a possible contributing factor to the results. To accomplish this, we examined linear and quadratic associations with age for all variables. To assess associations with age, we conducted Goodness-of-Fit analyses on all variables both linearly and quadratically. Linear vs. quadratic fit was not assessed by subtypes due to the smaller sample size.

## 3. Results

We report results by structure (cortical GM volume, corpus callosum, cerebellum cortex, thalamus, caudate, putamen, pallidum, hippocampus, and amygdala) and lobe (frontal, parietal, temporal, occipital). In these analyses, we compared FASD vs. controls and PAE/ARND vs. pFAS/FAS vs. controls.

The analysis included 123 participants (68 controls and 57 FASD) who ranged in age from 3 to 21 years of age. The sample was well matched on age across the age spectrum (see Figure 1; *p* = 0.63), use of 12- vs. 32-channel head coil (*p* = 0.79), and reported sex (*p* = 0.76). The control sample included a diverse representation of the local demographics with 42% Hispanic and 10% Native American. The FASD group represented the demographics of the FASD outreach clinics with 63% Native American and 20% Hispanic. Both race and ethnicity were significantly different by group (*p* < 0.05). As expected, IQ scores in the FASD group were lower than the control group (*p* < 0.001) and both FASD subtypes were significantly lower than controls (*p* < 0.001) but not different from each other (*p* = 0.36). There was no significant group or subtype difference in GPB time to completion (*p* > 0.05). More details on the study population are provided in Table 2.

**TABLE 2:** Demographics.

Measure	Controls (*N* = 68)	FASD (*N* = 55)	PAE (*N* = 8)/ ARND (*N* = 15)	pFAS (*N* = 7)/ FAS (*N* = 25)
Age (years)	11.1 (0.54)	10.7 (0.61)	10.8 (0.94)	10.7 (0.80)
Head coil (%12/32)	51.5/48.5	50.9/49.1	56.5/43.5	46.9/53.1
Reported sex (M/F)	34/34	26/29	11/12	15/17
Race (%)*†				
American Indian	10.3	63.6	73.9	59.4
Asian	5.9	1.8	0	3.1
African American	1.5	1.8	0	3.1
Caucasian	79.4	27.3	21.7	31.3
Unreported	2.9	3.6	4.3	3.1
% Hispanic*†	42.7	20	17.4	21.9
IQ*†	107 (2)	80 (2)	82 (3.5)	78 (2.8)
Grooved Pegboard	73.4 (3.0)	83.18	78.7 (5.9)	87.3 (5.7)

*p < 0.05 controls different than FASD, †*p* < 0.05 controls different than FASD subtypes.

### 3.1. Comparison of total brain volume, cortical thickness, and surface area between FASD and control participants. Covariates: head coil (12- vs. 32-ch)

As shown in Tables 1, 3, children with FASD had significantly reduced total brain volume and reduced volume in each of the individual structures assessed (corpus callous, cerebellum, thalamus, caudate, putamen, pallidum, and hippocampus). When controlling for eTIV, there was additional loss of volume beyond that expected based on the smaller head size in cerebellum, thalamus, caudate, pallidum, and hippocampus. There were no significant interactions between hemisphere and group. Similarly, there were main effects of sex, which in all cases revealed greater volume in males vs. females. However, there were no significant interactions between sex and group. For the lobar analyses, eTIV was included as a covariate. After correcting for multiple comparisons, significant group effects were seen in frontal and temporal GM volume and temporal lobe GM thickness with FASD less than controls in all cases.

**TABLE 3:** Statistical results for volume comparing controls to FASD.

	Group *F*(1,120) *p*-value	Sex *F*(1,120) *p*-value	Group (eTIV) *F*(1,119) *p*-value
Brain seg w/o ventricles	27.46 0.000025	37.97 0.000025	–
eTIV	18.88 0.000075	32.35 0.000012	–
Cortical GM	23.08 0.000018	24.57 0.000017	4.39 n.s.
Corpus callosum	14.63 0.00048	6.73 0.027	2.42 n.s.
Cerebellum cortex	45.93 0.000008	21.67 0.000033	23.37 0.000018
Thalamus	29.78 0.000006	21.74 0.00004	9.45 0.0067
Caudate	59.15 0.0000049	3.48 n.s.	35.8 0.000009
Putamen	20.35 0.000047	13.47 0.0011	n.s.
Pallidum	38.18 0.000012	17.02 0.00024	16.66 0.00024
Hippocampus	25.10 0.000008	12.54 0.0028	6.91 0.018
Amygdala	20.30 0.000044	23.15 0.000025	3.93 n.s.
Frontal GM	6.88 0.018	0.002 n.s.	
Parietal GM	0.21 n.s.	0.96 n.s.	
Temporal GM	7.97 0.011	0.3 n.s.	
Occipital GM	3.64 n.s.	1.24 n.s.	

n.s., not significant.

### 3.2. Comparison of total brain volume, cortical thickness, and surface area between FASD and control participants: goodness-of-fit analyses by age, linear vs. quadratic

Potential group differences in associations with age suggest reported group differences may be influenced by different developmental patterns. Linear vs. quadratic fit are noted in Table 1. Only fits that revealed statistical significance are noted. Cases that did not fit either model are noted as n/a. There were seven structures (corpus callosum, cerebellum, putamen, pallidum, hippocampus, parietal volume, and occipital volume) that revealed different patterns between controls and FASD across age.

### 3.3. Comparison of total brain volume, cortical thickness, and surface area between PAE/ARND, pFAS/FAS, and control participants. Covariate: head coil (12- vs. 32-ch)

**FIGURE 3:**
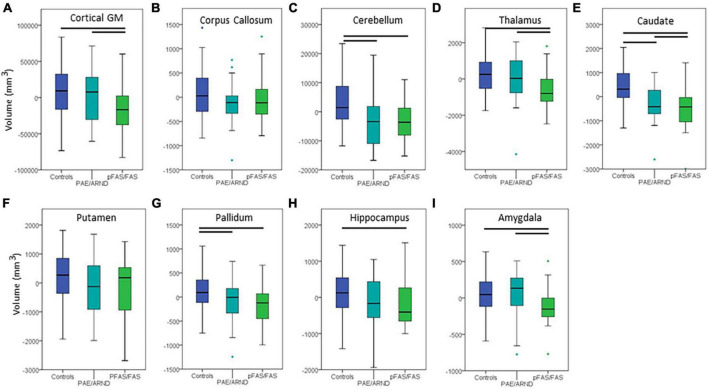
Differences in cortical volume between controls and FASD subtypes: **(A)** Cortical GM, **(B)** corpus callosum, **(C)** cerebellum, **(D)** thalamus, **(E)** caudate, **(F)** putamen, **(G)** pallidum, **(H)** hippocampus, and **(I)** amygdala. All data represent the residuals taking into account the covariates of coil channel number and eTIV. The most common pattern observed is consistent with panel **(A)**. Total cortical GM volume in which controls and the PAE/ARND subtype had greater volume than the pFAS/FAS subtype. However, a few structures revealed a significant reduction in volume in both subtypes, as in panel **(C)** cerebellum. In some cases, group differences were only significant between controls and pFAS/FAS subtype with no significant difference of PAE/ARND from either group, as in panel **(H)** hippocampus. A few of the examined structures revealed no group differences, such as **(F)** putamen after including eTIV as a covariate.

**FIGURE 4:**
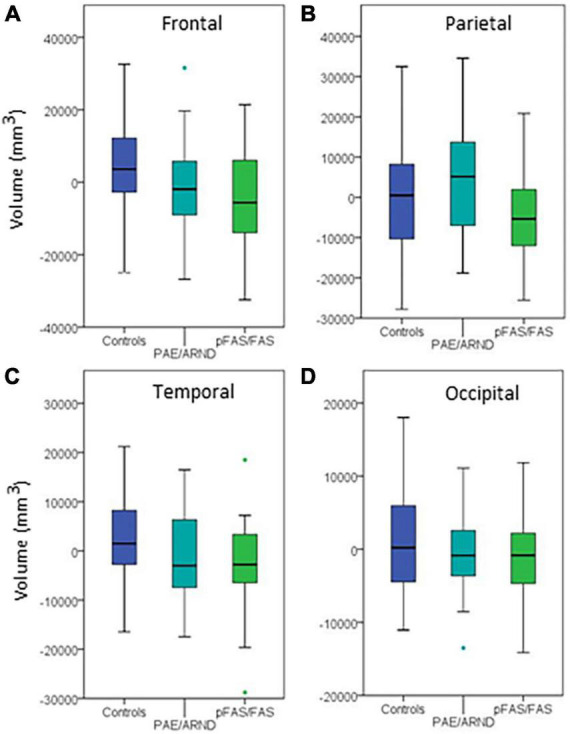
Regional differences in cortical volume between controls and FASD subtypes: **(A)** Frontal lobe, **(B)** parietal lobe, **(C)** temporal lobe, and **(D)** occipital lobe. Although three of the regions showed group differences before controlling for eTIV, none revealed significant differences when controlling eTIV after multiple comparisons correction.

Subtype statistical analyses are presented in Table 4 and example data from different structures are presented in Figures 3, 4. There was a significant difference in overall brain volume when comparing controls vs. the two FASD subtypes with *post hoc* analysis demonstrating the pFAS/FAS group was significantly smaller than PAE/ARND and controls, with no significant difference between PAE/ARND vs. controls. Individual structures revealed significant differences between the three groups, similar to the group analysis described above. In most cases, the pFAS/FAS group values were significantly smaller than the other two groups. However, there were some notable exceptions. For cerebellum, caudate, and pallidum, controls had significantly greater volume than both FASD subtypes, and the PAE/ARND subtype had greater volumes than the pFAS/FAS subtype. When eTIV was included as a covariate the following structures continued to reveal group differences above and beyond those expected based on the differences in overall brain volume: cortical GM volume, cerebellum, thalamus, caudate, pallidum, hippocampus, and amygdala. There were no significant interactions between hemisphere and group.

**TABLE 4:** Statistical results for volume controls vs. PAE/ARND vs. pFAS/FAS.

	Group *F*(2,118) (*p*-value)	Sex *F*(1,118) (*p*-value)	Group (eTIV) *F*(1,117) (*p*-value)	*Post hoc* comparisons†
Brain seg w/o ventricles	29.3 (0.000025)	37.85 (0.000025)	–	C,A>F
eTIV	23.7 (0.000012)	33.6 (0.000012)	–	C,A>F
Cortical GM	27.38 (0.000008)	27.09 (0.000008)	4.15 (0.03)	C,A>F (C,A>F)
Corpus callosum	10.57 (0.0001)	7.59 (0.017)	1.5 (n.s.)	C,A>F
Cerebellum cortex	32.59 (0.000005)	24.87 (0.000001)	12.21 (0.000075)	C>A>F (C>A,F)
Thalamus	34.07 (0.000004)	26.23 (0.000006)	8.29 (0.0011)	C,A>F (C,A>F)
Caudate	46.6 (0.0000035)	2.32 (n.s.)	20.18 (0.000009)	C>A>F (C>A>F)
Putamen	18.48 (0.000004)	12.41 (0.0019)	2.09 (n.s.)	C,A>F
Pallidum	33.0 (0.000003)	15.78 (0.00042)	9.59 (0.0047)	C>A>F (C>A,F)
Hippocampus	20.4 (0.000006)	12.02 (0.002)	3.38 (0.046)	C,A>F (C>F)
Amygdala	25.6 (0.000002)	22.38 (0.00002)	4.84 (0.02)	C,A>F (C,A>F)
Frontal GM	5.94 (0.031)	0.18 (n.s.)		C>F
Parietal GM	4.2 (0.03)	1.97 (n.s.)		C,A>F
Temporal GM	4.07 (0.033)	0.65 (n.s.)		C>F
Occipital GM	2.5 (n.s.)	1.96 (n.s.)		

C, controls, A-PAE/ARND group, F-pFAS/FAS group. n.s., not significant. †Significant *post hoc* comparisons for group without and with eTIV (in parentheses) included as a covariate after accounting for multiple comparisons.

Regional analysis revealed significant group differences in frontal, parietal, and temporal lobe GM volume with controls generally revealing significantly greater volume than the pFAS/FAS group. Furthermore, there was a significant difference in temporal lobe GM thickness with controls having greater CT than the pFAS/FAS group. Finally, there was a significant difference in parietal lobe SA with differences only revealed between PAE/ARND vs. pFAS/FAS groups. In this case, the PAE/ARND group had greater SA than controls and pFAS/FAS, although the difference was only significant between the FASD subtypes.

### 3.4. Correlation between brain structure and IQ and GPB measures

**FIGURE 5:**
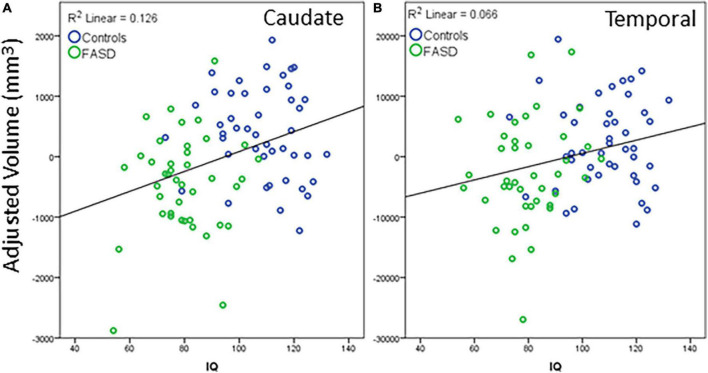
Example correlations of IQ scores with cortical volume: **(A)** Caudate and **(B)** temporal lobe. The data represent the residuals with covariates of age, eTIV, and channel number regressed out. All significant correlations revealed a similar positive correlation between structure volume and IQ scores. Blue dots are controls, green dots are FASD.

Regions that revealed a significant group difference were evaluated for correlations with behavioral measures. Correlations were examined across the full sample. Age was included as a covariate in all analyses and left and right hemisphere values were summed to create total volume for structures that did not reveal a significant effect of hemisphere. IQ score was highly correlated with all brain structural measures assessed (see Table 5). When including eTIV as an additional covariate, a significant correlation with IQ score remained for cerebellum, left and right caudate, and left pallidum (see example correlations in Figure 5). There were no regions that revealed a significant correlation with GPB following correction for multiple comparisons.

**TABLE 5:** Correlations between IQ and brain volume adjusted for age, eTIV and channel number.

	IQ *r* (*N* = 86) (*p*-value)
Total GM volume	0.26 (n.s.)
Corpus callosum	0.057 (n.s.)
Cerebellum	0.259 (n.s.)
Thalamus	0.182 (n.s.)
Caudate	0.355 (0.003)
Putamen	0.194 (n.s.)
Pallidum	0.241 (n.s.)
Hippocampus	0.106 (n.s.)
Amygdala	0.048 (n.s.)
Frontal lobe	0.263 (n.s.)
Parietal	0.095 (n.s.)
Temporal	0.257 (0.042)
Occipital	0.176 (n.s.)

n.s., not significant.

## 4. Discussion

We hypothesized that the FASD group would show smaller brain volume, reduced CT, and reduced SA compared to controls and that PAE/ARND subtype would reveal intermediate values between controls and the pFAS/FAS subtype. Consistent with this hypothesis, the FASD participants presented with significantly smaller brain volume compared to the control participants. However, in many cases, individuals with the PAE/ARND subtype did not differ significantly from controls, revealing volumetric differences present only in the pFAS/FAS subtypes of FASD. However, participants with pFAS/FAS and PAE/ARND had similarly decreased volume compared to the control group in other regions, and the controls presented with significantly increased volume in the caudate, cerebellum, and pallidum structures compared to both FASD groups. Participants with FASD had significantly decreased total SA compared to the controls, specifically in the frontal region. Goodness-of-Fit analysis by age with linear and quadratic models indicated the fit between linear and quadratic models for both the FASD and control participants showed mixed results. This indicates differences in developmental patterns may partially explain the group differences in an otherwise well-matched sample.

The results of this large single-site study are consistent with studies that used sMRI to document reduced sizes of the corpus callosum, caudate, and eTIV in individuals with FAS relative to the other FASD subtypes (Archibald et al., 2001; Riley et al., 2004; Inkelis et al., 2020; Krueger et al., 2020) and provide partial replication of prior findings in an independent sample with a diverse representation.

The results reported here are consistent with prior findings when comparing FASD and control groups. Archibald et al. (2001) reported a significant decrease in caudate volumes in the FAS group compared to the control group. Consistent with Inkelis et al. (2020), in a study of adolescents and young adults (13–30 yrs), brain volume reductions were evident in adulthood in corpus callosum and caudate (reported volume decreases in adulthood). Jacobson et al. (2017) reported a significant decrease in corpus callosum volume in infants heavily exposed to prenatal alcohol among the FAS group relative to control groups irrespective of age and sex. Krueger et al. (2020) reported decreased caudate and intracranial volumes in a PAE group compared to controls with no significant differences in age and sex. These results underscore the teratogenic effects of prenatal alcohol irrespective of age of assessment or sex.

Our lobar analysis results showed significant group differences in frontal, and temporal lobes with greater volumes in the controls compared to FASD group. Regional analyses indicated that inferior parietal/perisylvian regions and the anterior and orbital frontal cortex are significantly reduced in individuals with PAE relative to controls (Archibald et al., 2001; Sowell et al., 2002a). However, parietal lobes showed greater reductions than frontal and temporal lobes in other studies (Archibald et al., 2001; Riley et al., 2004; Fernández-Jaén et al., 2011). The current results are not consistent with the prior findings demonstrating the greatest effect in parietal lobe. This may be related to smaller sample size in prior studies and the current results indicating a regression to the mean with larger samples or differences in drinking patterns which may differ by site (May et al., 2018).

Limited differences in CT are consistent with the differences in temporal lobe volume with decreases in temporal GM thickness in the FASD group relative to controls. Prior results on CT are mixed with small clinical samples (Treit et al., 2014). A rat model of PAE (Leigland et al., 2013) revealed cortical thinning, whereas a sample of individuals with PAE were reported as having increased CT (Fernández-Jaén et al., 2011). More recent multi-site studies have demonstrated robust group differences with reduction in CT with PAE (Zhou et al., 2018). Other results reveal different developmental patterns in CT across childhood (Zhou et al., 2011). This variability in results in CT across studies could be attributed to small samples, differences in the amount or patterns of maternal alcohol consumption, synaptic development and myelination related to age effects, imaging protocols, statistical analysis, differences in demographics, and differences in the age of the cohorts across studies.

PAE-related SA decreases are reported in both human and animal models [(Leigland et al., 2013) - mice; (Migliorini et al., 2015) - PAE; (Rajaprakash et al., 2014) – ARND). Our current study revealed differences in the parietal lobe SA only with the PAE/ARND subtype having greater SA when controlling for eTIV relative to both the controls and the pFAS/FAS. Sowell et al. (2002b) reported a reduced SA in participants with an FASD in the orbital frontal cortex. The SA analysis performed by Migliorini et al. (2015) was limited to only the anterior cingulate cortex and found decreased SA in the sample of heavy PAE exposed individuals relative to controls. Since brain volume is a combination of CT and cortical SA and we found no differences in CT in parietal lobe, the current results indicate that volumetric differences in parietal lobe are more likely related to SA changes rather than CT.

An important consideration in comparing results across studies in childhood is that brain structure changes with development. That is, cortical thinning in adolescence is expected as a part of typical development. Inkelis et al. (2020) showed a quadratic relationship with age among the FASD group compared to controls. Though inconsistent across structures, the current results revealed different developmental patterns (linear vs. quadratic) between controls and FASD groups as a function of age. These group differences in brain volume indicate that results may differ as a function of age and must be considered when determining if results replicate across samples.

The primary contribution of the current analysis was the examination of the effect of FASD subtype on brain structure. The pattern of results differed by structure with some structures revealing that only the pFAS/FAS group differed from controls, whereas volume was smaller than controls in both subtypes in other structures. While FASD subtype does not necessarily equate to dose effects, finding consistent differences in both subtypes supports the idea that certain brain regions are more susceptible to PAE than other structures. This sensitivity may be related to different patterns of drinking instead of a direct link to amount of alcohol consumed. This result demonstrates the importance of examining volumetric differences by FASD subtype to fully understand the effects of PAE on brain growth parameters. Most of the prior results have not examined results relative to FASD subtype likely due to limited sample size.

Overall IQ scores were highly correlated with all the structures assessed (see Table 4) with cerebellum, left and right caudate, and left pallidum revealing significance when controlling for age and eTIV. The significance of the correlations without controlling for eTIV is expected given the group differences in IQ and the group differences in cortical volume. The structures, which remain significant when controlling for eTIV, reveal that subcortical structures are relevant to overall estimates of intelligence. These results are similar to those of Gautam et al. (2014), which revealed a positive correlation between IQ scores and white matter volume changes with increasing age in the FASD participants relative to controls. Hendrickson et al. (2018) and Zhou et al. (2018) reported no correlation between IQ and regional brain volume, which is consistent with the current results showing no relationship with parietal, frontal and occipital lobar volumes when controlling for eTIV.

A strength of the current study is the almost equal representation of males and females and the equal distribution of FASD subtypes and control groups across age. Additionally, the participants were recruited from one site, which likely reduced variability in diagnostic criteria and variability in scanner protocols relative to prior multi-site studies (Roussotte et al., 2012; Yang et al., 2012a; Treit et al., 2016; Zhou et al., 2018). Despite being relatively large, the FASD sample size remains a limitation of the current study due in part to the inherent variability in alcohol exposure that is present in human studies of FASD. Due to the limitations in alcohol biomarkers to independently assess drinking during pregnancy and a lack of consistent tracking of alcohol consumption during pregnancy and during prenatal care, the samples from different studies may represent different overall drinking patterns *in utero* which may lead to variation across studies. Zhou et al. (2011) cited comorbidities and additional exposure to drugs as factors that may contribute to discrepancies in cortical thinning among participants with an FASD. Additional co-exposures were not tracked in the reported sample and should be incorporated into future studies. An additional limitation of the current study is that it is cross-sectional rather than longitudinal. Therefore, the differences in developmental patterns across age cannot be easily interpreted. Instead, the primary analysis focused on group differences on this well-matched sample across the age spectrum. This study examined sex as a variable of interest in line with recommendations (De Castro et al., 2016; Heidari et al., 2016). However, biological sex was parent-/self-reported and we did not inquire separately about gender. Future studies should capture biological sex at birth as well as current gender. Furthermore, assessing hormone levels will facilitate our understanding of how PAE influences brain development in the presence of different hormone levels (Fung et al., 2020). Finally, all participants in the FASD group were recruited from the FASD clinic and do not represent a community sample of children with PAE. Future large-scale studies that include longitudinal assessments, additional pre- and post-natal exposure data, and a community sample of children with PAE may bring clarity to the variability in results.

The current study reinforces the teratogenic effects of PAE resulting in abnormalities in brain volume and SA in children and adolescents with PAE. The results of the study are consistent with previous findings with smaller sample sizes and studies with large sample sizes derived from multisite cohort samples. The current study uniquely captured data solely from one site with a comparatively large sample. Despite total brain volume indicating that children with PAE/ARND are statistically equivalent to controls, analysis of key brain structures reveal patterns that indicate similar levels of brain damage in both FASD subtypes. Unlike past studies that have indicated that dysmorphology is related to greater structural abnormalities, the current results indicate that some structures are sensitive to PAE at an equivalent level in the absence of facial dysmorphology. The variation in results by FASD subtype may help explain why individuals with ARND perform similarly to children with FAS/pFAS on certain cognitive tasks. These results underline the importance of minimizing PAE to reduce the long-term effects on brain development.

## Data availability statement

The raw data supporting the conclusions of this article will be made available by the authors, without undue reservation.

## Ethics statement

The studies involving human participants were reviewed and approved by the University of New Mexico Health Sciences Center Human Research Review Committee. Written informed consent to participate in this study was provided by the participants’ legal guardian/next of kin.

## Author contributions

JS and DH contributed to the conception and design of the study. CC and DH oversaw participant assessment and reporting of neuropsychological testing results. KB, CR-H, JP, and JS extracted the data and performed the statistical analysis. TB created the first draft of the manuscript. TB, KB, FT, KV, and JS wrote the sections of the manuscript. All authors contributed to the manuscript revision and read and approved the submitted version.

## Footnotes

1
http://surfer.nmr.mgh.harvard.edu/
